# Biomarkers in asthma: state of the art

**DOI:** 10.1186/s40733-018-0047-4

**Published:** 2018-12-21

**Authors:** Angelica Tiotiu

**Affiliations:** 1 Pulmonology Department, University Hospital, 9, Rue du Morvan, 54511 Nancy, Vandœuvre-lès-Nancy France; 2 EA 3450 DevAH, Development, Adaptation, Cardio-Respiratory Regulations and Motor Control, University of Lorraine, Nancy, France; 3 National Heart and Lung Institute, Airway Disease Section, Imperial College London, London, UK

**Keywords:** Asthma phenotypes and endotypes, Personalized medicine, Biomarkers, Targeted therapy

## Abstract

Asthma is a heterogenous disease characterized by multiple phenotypes driven by different mechanisms. The implementation of precision medicine in the management of asthma requires the identification of phenotype-specific markers measurable in biological fluids. To become useful, these biomarkers need to be quantifiable by reliable systems, reproducible in the clinical setting, easy to obtain and cost-effective.

Using biomarkers to predict asthma outcomes and therapeutic response to targeted therapies has a great clinical significance, particularly in severe asthma. In the last years, significant research has been realized in the identification of valid biomarkers for asthma. This review focuses on the existent and emerging biomarkers with clinical higher applicability in the management of asthma.

## Background

Asthma is a heterogeneous disease diagnosed by the presence of intermittent symptoms of wheeze, cough and chest tightness, typically related to a reversible airflow obstruction, usually resolves spontaneously or with asthma treatment [1, 2]. Over the years, clinicians have defined several phenotypes based on the presentation and age of onset of symptoms, the severity of the disease, and the presence of other conditions such as allergy and eosinophilia with different long-terms outcomes and response to therapy with corticosteroids [2]. Despite the recognition of these phenotypes of asthma, the approach to the management of asthma recommended by the international Global Initiative for Asthma (GINA) guidelines continues to be based on the severity of the condition, with drugs added on the basis of asthma control [2].

In the era of the personalized medicine, in order to deliver this approach for asthma, it is important to be able to phenotype the condition in an unbiased way and to define biomarkers able to predict the course of the disease and the response to therapy [2, 3]. A biomarker is a measurable indicator that can evaluate a normal or pathological biological processes or pharmacologic response to a therapeutic intervention [2]. A valid biomarker would have several key characteristics: to distinguish between disease and health with high positive and negative predictive values, to provide information about disease prognosis and clinical outcomes, to change with disease progression and “normalize” with successful treatment, to be reliable and reproducible in the clinical setting with little or no day-to-day variation, to be easy to collect in the “real-world” setting, to be quantifiable in an analytical system with well-defined performance, and to be cost-effective [4, 5].

Despite the sustained research efforts during the last years focused on the identification of biomarkers applicable in clinical practice for the management of asthma, only a few biomarkers indicative of T2-high asthma have been described (e.g. IgE, eosinophils in blood and/or sputum, Fractional Exhaled Nitric Oxide [FeNO], periostin), and their utility in diagnosis, prognosis and therapy is still controversial [3, 6].

This review will summarize the recent knowledge about the biomarkers (proteins and related substances) identified of asthma with special focus on those with higher clinical applicability.

## Biomarkers

### Blood cells and serum biomarkers

Using the blood for requiring biomarkers is micro-invasive (the procedure can be painful and difficult in some patients) and easy to realize in the clinical setting, requires minimal patient effort, could be collected across the age spectrum, and it is cost-effective [4].

*Blood eosinophil count* is not useful for the diagnosis of asthma (GINA), but it can serve as prognostic biomarker and to predict several therapeutic responses [3] in asthmatic patients with type 2 inflammation.

A recent study realized on a large cohort in UK, showed that patients with blood eosinophil counts greater than 400 cells/μL experienced significantly more severe exacerbations (adjusted rate ratio RR 1·42) and acute respiratory events (RR 1·28) than those with counts of 400 cells/ μL or less and had significantly lower odds of achieving overall asthma control (odds ratio OR 0·74) [7]. Another study found that blood eosinophilia (> 400 cells/ μL) is a risk factor for airflow obstruction in asthmatic patients (even in those without symptoms) and predicts an enhanced longitudinal decline in lung function, independently of smoking status [8].

Similarly, in a pediatric cohort [9], blood eosinophilia (≥ 300 cells/μL) is associated with asthma severity (*p* = 0.036), high atopy (*p* = 0.001), more exacerbations (*p* = 0.022), FEV1/FVC (*p* = 0.004), and bronchial hyperresponsiveness (*p* = 0.002).

Blood eosinophils counts can predict responsiveness to corticosteroid therapy. In atopic asthmatic children with blood eosinophilia (≥ 300 cells/μL), daily inhaled corticosteroids use is associated with more asthma control days and lower exacerbations rate [10]. Previous data showed that blood eosinophils count could be useful to monitor the response to oral corticosteroids because the adjustment of dose to maintain blood eosinophilia < 200 cells/ μL was successful in preventing exacerbations, improving asthma control and resulted in less prednisone used [11]. The up-dosing of inhaled corticosteroids is associated with a decrease in blood eosinophilia [12] but studies about the interest to monitor blood eosinophils to adapt the dose of inhaled corticosteroids (ICS) to maintain asthma control are lacking for the moment.

Baseline blood eosinophils count is used as biomarker to predict the clinical efficacy of biological therapies as anti-IL5 antibody (mepolizumab, reslizumab), anti-IL5 receptor antibody (benralizumab) and anti-IL4 receptor antibody (dupilumab) [13–16]. The cut-off of blood eosinophils count is 300 cells/μL for most biologics, except the reslizumab (400 cells/μL). In these trials, patients with eosinophilia responded better to biologics therapies.

Previous data suggested that blood eosinophilia (≥ 300 cells/μL) is associated with greater response to anti-Ig E antibody (omalizumab) [17, 18], but this result was not confirmed by a recent real-life study [19].

Although the blood eosinophil count is easy to obtain and correlates well with sputum eosinophilia [20–22], the problem is that the optimal cut-off has yet to be established and its levels may be elevated due to co-existing conditions such as parasitic infestations, thus limiting its use as a predictive biomarker.

Currently, *blood neutrophils count* is not a biomarker for asthma diagnosis (GINA) but a recent study [23] showed that neutrophilia may differentiate between patients with a smoking history (≥10 pack-years) and adult onset asthma from those with asthma-chronic obstructive pulmonary disease overlap syndrome (ACOS) (3850 cells/ μL vs 4500 cells/ μL, *p* = 0.008). ACOS patients had a persistent airflow limitation, a lower diffusing capacity of the lungs for carbon monoxide then other patients and a higher number of comorbidities (Hypertension, Coronary heart disease, Hypercholesterolemia) [23].

The EGEA2 study found that persistent blood neutrophilia ≥5000/ μL was associated with poor symptom control (OR 3.09) and increased exacerbations suggesting that increased blood neutrophils count could be a prognostic biomarker [24].

Peripheral differential cell counts may reflect the airway inflammation. A meta-analysis of 14 studies showed an area under the curve (AUC) of 0.78 for blood eosinophils, high predictive for airway eosinophilia in contrast with blood neutrophilia AUC of only 0.6, less indicative of sputum neutrophilia [25].

*Eosinophil cationic protein (ECP)* is found in the primary matrix of the eosinophil and is released during the eosinophil degranulation. Previous data found that the serum ECP is increased in adults and children with atopic asthma, associated with airway resistance and bronchospasm [26]. Similarly, serum ECP concentration is increased in children with asthma during an exacerbation [27] and normalize with the decrease of airway resistance value after 8 weeks of treatment by montelukast [28]. As predictor of therapeutic response, one study in a pediatric population showed that higher baseline serum ECP level was associated with greater improvement in lung function after ICS treatment [28]. It has been suggested that ECP assessment could be useful for the initiation and dose titration of ICS in younger children in whom other biomarkers might be less feasible to asses [4], but other complementary studies are needed to validate this strategy.

*Periostin,* an extracellular matrix protein secreted by airway epithelial cells in response to IL-13 that regulates epithelial-mesenchymal interactions [4], has been associated with T2-high eosinophilic asthma [29]. Periostin expression is increased in the asthmatic airway [30] and may be measured in the serum [31]. At the moment, the concordance between serum periostin concentration and sputum eosinophilia has not been well established with contradictory results [21, 32]. The periostin plays key roles in bone growth, and in children at 2 years of age, serum periostin levels were up to 2- to 3-fold higher than previously observed adult levels [33]. The same study showed that the level of periostin at 2 years of age was predictive of asthma at age 6 years old [33]. The stability of serum periostin over disease progression in adults with asthma (without seasonal effect) [34] and in children between 4 and 11 years of age, supports its use as a biomarker for type 2-high asthma. Previous data found that elevated levels of serum periostin in adults with asthma are associated with fixed and more severe airflow obstruction [35, 36], and greater lung function decline [37, 38]. Several studies showed that the elevated serum periostin level predicts the response to omalizumab therapy [35, 39].

*Lipoxins* have anti-inflammatory action and play an important role in chemotaxis and related signal transduction [4]. In patients with severe asthma, lipoxin A_4_ expression is decreased in the airways [40] and systemic circulation [41], associated with decreased expression of related enzymes and receptors necessary for lipoxin biosynthesis [40, 42] and persistent innate lymphoid cell (ILC) activation and eosinophilia [43]. In severe asthma, the expression of lipopolysaccharide-stimulated lipoxin A_4_ biosynthesis in airways macrophages is decreased and strongly associated with the degree of airflow obstruction [44]. The mechanism of lipoxin A_4_ suppression in severe asthma are unclear but could be related to systemic corticosteroid treatment or to oxidative stress [4, 42]. Inhibitors of soluble epoxide hydrolase increased lipoxins levels that mediated antiphlogistic actions, suggesting a new possible therapeutic approach for severe asthma [42, 45].

*IgE* is an immunoglobulin which mediates type 1 hypersensitivity reactions and plays a key role in the pathogenesis of allergic asthma. It binds to IgE receptors on mast cells and basophils, producing cytokines that mediate T2 responses [46]. Serum Ig E closely correlates with the risk of asthma [47]. Previous data in pediatric cohorts showed that higher serum Ig E is associated with atopy (increased aeroallergens sensitisation), airway hyperresponsiveness (AHR), bronchial wall thickening, and more severe asthma [9, 48, 49]. A significant inverse association was found previously between total serum IgE and FEV1/FVC independently of smoking and asthma status in a longitudinal evaluation in general population [50]. Total serum IgE does not predict the response to omalizumab, despite this molecule being not only the drug target, but also the basis for its dose calculation [51]. A recent prospective study showed that the reduced free serum IgE levels from baseline after 16–32 weeks of treatment by omalizumab were associated with reduced exacerbation numbers at 2 years [39].

*Chitinases* are hydrolases characterised by their affinity to cleave chitin that are thought to play a role in remodelling and regulation of the extracellular matrix [4]. The chitinase-like protein YKL-40 (human cartilage glycoprotein 39) same to be an interesting biomarker for distinguishing asthma from chronic obstructive pulmonary disease (COPD) and healthy controls [52], as well between patients with ACOS and COPD [53]. Detectable in the serum and airways, associated with subepithelial basement membrane thickness in both adults and children, YKL-40 level correlates with severe asthma and irreversible airway obstruction [9, 54]. YKL-40 expression is increased during asthma exacerbations [55], and could predict longitudinal decline of lung function in response to cigarette smoke exposure [56]. More studies are needed to prove how useful YKL-40 is in the assessment of future asthma outcomes and risk.

Recent data showed that *CCL26* is the best discriminator for type 2 inflammation [57], *serum urokinase plasminogen activated receptor* is elevated in adult patients with severe, non-atopic asthma [58], and the expression of ten selected *microRNA* (HS_108.1, 112, 182.1, 240, 261.1, 3, 55.1, 91.1, has-miR-604, and has-miR-638) is higher in children with severe asthma [59]. *Serum high sensitive C-reactive protein (hs-CRP)* is increased in asthmatic patients than in healthy control, in poorly controlled vs well controlled, and may be a useful biomarker of airway inflammation in non-smoking asthmatic patients without complications, such as heart disease, hypertension, hyperlipidaemia, chronic obstructive pulmonary disease, or infection [60, 61]. Evaluation of inflammatory markers *interleukin-6 (IL-6)* and *matrix metalloproteinase-9 (MMP-9)* in serum showed higher levels in asthmatic patients vs controls and were associated with a more severe asthma [62]. A high serum level of *IL-8* could discriminate COPD from asthma patients [63].

Although all advantages of serum biomarkers, it is important to remember that peripheral blood studies often do not reflect airway biology, and therefore peripheral blood biomarkers might not represent physiologic mechanisms in the airways [29].

### Sputum cells and mediators

Induced sputum is a non-invasive method which allows to quantify the inflammatory cell pattern in airways of asthmatic patients [4, 46]. To obtain samples for sputum analysis, patients nebulize 3% saline for 20 min and the sputum expectorated over this period is centrifuged, stained, and analysed by quantifying the number of different cell types [46].

*Sputum quantitative cell count* is the reference standard to reflect the airway inflammation in asthma. The practical advantage of sputum differential cell counts is that this method is feasible even on frozen samples [3]. Four inflammatory phenotypes have been identified in the Severe Asthma Research Program (SARP) cohort – eosinophilic (≥2% eosinophils in induced sputum), neutrophilic (≥40% neutrophils), mixed granulocytic and paucigranulocytic [64]. Unfortunately, the cut-off used to define the sputum eosinophilia and neutrophilia is different in the other cohorts of asthmatic patients: Airways Disease Endotyping for Personalized Therapeutics (ADEPT) and Unbiased Biomarkers for the Prediction of Respiratory Disease Outcomes (UBIOPRED) (≥ 3% eosinophils, respectively 60% neutrophils) [65]. Sputum analysis of the UBIOPRED cohort identified 3 transcriptome-associated clusters (gene clusters), corresponding to eosinophilic, neutrophilic, and paucigranulocytic phenotypes [66]. A six-gene signature (CLC, CPA3, DNASE1L3, IL1B, ALPL, and CXCR2) can differentiate asthma patients from controls, discriminate inflammatory phenotypes of asthma and predict the ICS response [67], but this method is not currently available.

The presence of sputum neutrophilia is one candidate predictive biomarker for non-T2 asthma [46]. Previous data evaluating sputum inflammatory patterns in patients with asthma showed that 20% had sputum neutrophil percentages of > 61% [68]. Sputum neutrophilia is associated with asthma severity and poor response to corticosteroids [64, 69]. Macrolide treatment could be a possible therapeutic intervention for these patients. Clarithromycin administration (500 mg twice daily) in patients with refractory asthma reduced the airway neutrophil counts and improved the quality of life in patients undergoing active treatment. A subgroup analysis in patients with sputum neutrophilia of > 61% showed that they had greater improvements in quality of life scores compared with those without sputum neutrophilia [70]. A more recent trial (AMAZES) [71] confirmed the benefice of the macrolide treatment with a reduction in exacerbation rate and an improvement of quality of life in patients with refractory asthma who took azithromycin 500 mg three times per week for 48 weeks. Prior data suggested that activation of CXCR2 resulted in increased airway neutrophilia, thus contributing to the pathogenesis of non-eosinophilic asthma, but a recent trial with a CXCR2 antagonist in severe neutrophilic asthma (sputum neutrophils > 40%) not showed a significant improvement in asthma outcomes despite the reduction of sputum neutrophilia [72].

Recent data found that changes in sputum eosinophil count over time reflect fluctuations in clinical asthma control [73]. The high level of Group 2 ILC in the sputum is corelated with severe asthma whose airway eosinophilia is greater than 3%, despite normal blood eosinophil numbers (< 300/μL) suggesting these cells could be a potential novel biomarker [74].

Sputum eosinophilia ≥3% predicts response to corticosteroids [75]. Targeting a sputum eosinophil level in adult asthmatics of 1–3% reduced exacerbation rates as compared to usual care [76, 77]. Current treatment guidelines for severe asthma recommend using sputum eosinophil counts to adjust corticosteroid treatment in centres that have experience with this laboratory technique [78]. Subsequent work has also identified sputum eosinophilia not only as a validated biomarker for corticosteroid therapy, but also as a biomarker for biotherapies [46]. Anti-IL 5 monoclonal antibodies (mepolizumab, reslizumab) improved quality of life and decreased exacerbation rate in patients with sputum eosinophilia of greater than 3% [79, 80]. Dupilumab, a targeted therapy against IL-4R-alpha that modulates the IL-4/IL-13 pathway, improved asthma control and lung function in asthmatic patients with sputum eosinophilia (≥3%) or blood eosinophilia (≥300/μL) [81]. A trial for fevipiprant, an antagonist of the prostaglandin-D2 receptor, enrolled patients with a sputum eosinophil count ≥2% show a reduction in sputum eosinophilia in treated patients [82].

Unfortunately, despite its use as a biomarker in many clinical trials, the use of sputum cells count in daily practice has limitations. This method requires specialized training, equipment, and laboratory for processing, patient coaching and cooperation, emergency protocols and equipment, is difficult to collect (impossible in young children), not easily repeatable, and had several contraindications [4, 46].

Several *sputum mediators* could be the potential biomarkers. For the diagnostic of inflammatory pattern, *sputum eosinophil peroxidase* is correlated with sputum eosinophilia [72], *specific microRNAs* could discriminate neutrophilic from eosinophilic asthma [83], and *neutrophil myeloperoxidase* has the potential to differentiate ACOS from asthma [84]. As prognostic biomarker, sputum expression of human *tumor necrosis factor-like weak inducer of apoptosis* (TWEAK) correlates with higher severity, poor asthma control and decreased lung function in children with non-eosinophilic asthma [85].

### Exhaled breath analysis

Analysing of *exhaled breath condensate (EBC)* offers a noninvasive method of sampling the airway environment. It analyses both volatile and nonvolatile compounds by saturating exhaled breath with water vapor and collecting the condensed material [46]. Examples of compounds collected in EBC include nitric oxide products, hydrogen peroxide, leukotrienes, and cytokines. Several components correlate with asthma diagnosis, others with asthma severity [46]. Clinical practice guidelines exist which allow for standardized collection techniques [86]. Concentrations of exhaled hydrogen ions, nitric oxide products, hydrogen peroxide and 8-isoprostanes were increased and related to lower lung function tests in adults with asthma compared to healthy subjects [87]. A previous study showed that ICS decrease hydrogen peroxide level in expired air condensate in asthmatic patients [88]. The disadvantages of this method are the absence of a well correlation with samples obtained by BAL, and the difficulty to determine the concentration of a given component due to variable dilution for non-volatile components [87].

*Fractional nitric oxide in the exhaled breath* (FeNO) provides information about the inflammatory state of the airways [89]. Nitric oxide plays key roles in lung biology as bronchodilator and inflammatory mediator and is produced in the lung from nitric oxide synthases during the conversion of the amino acid L-arginine to L-citrulline [4]. The biomarker FeNO is originated from nitric oxide production by the airway epithelium as a result of inducible nitric oxide synthase upregulation during the process of allergic inflammation [4]. A level of less than 25 parts per billion (ppb) is normal in adults, and a level greater than 50 ppb is elevated; the American Thoracic Society guidelines did recommend that FeNO values from 25 to 50 ppb (20–35 ppb in children) be interpreted cautiously and with reference to the clinical context [89].

The FeNO displays an AUC of 0.8 for asthma diagnosis [3]. Very high or low cut-off for FeNO can rule-in, respectively rule-out asthma [90]. FeNO has the limited utility to predict sputum eosinophilia [25]. In both children and adults, FeNO correlated with greater airway hyperresponsiveness as well as the risk of exacerbation [9, 89, 91]. Previous data showed that high FeNO levels (≥50 ppb) are associated with current asthma symptoms, asthma attacks and asthma-related emergency department visits [92]. Elevated FeNO levels predict response to ICS [93].A systematic review found that using FeNO to guide ICS therapy in adults reduced the mild but not the severe exacerbations [94]. However, a study including adults’ patients with well-controlled mild-to-moderate persistent asthma found that FeNO-guided management was not superior to physician assessment-based adjustment of ICS treatment in the time to asthma treatment failure [95]. ICS typically suppresses FeNO levels, and thus measuring it serially can be useful as a marker of compliance among asthmatics [96].

FeNO has been used less often as a predictive biomarker in recent clinical trials with biotherapies. Patients with a FeNO ≥50 ppb had a positive response to mepolizumab [97] or benralizumab [98] therapy while a FeNO level ≥ 19.5 ppb is correlated with a response to omalizumab therapy [18]. In patients treated by dupilumab, the degree of reduction in the FeNO level during the treatment corresponded with the improvement in lung function confirming the biologic activity of the drug [81].

However, despite its capabilities (noninvasive technic, easy to collect in the clinical setting, with a minimal patient effort), the use of FeNO has some limitations. Normal values vary by age, height, and according to the type of analyser used. Other confounding factors include smoking, atopy, and the use of corticosteroid treatment [46]. FeNO as a single, stand-alone biomarker might not be particularly useful and should perhaps be used as part of a more comprehensive panel [4]. The current guidelines for the treatment of severe asthma do not recommend the use of FeNO in the routine for the management of adults and children with asthma [78].

The evaluation of exhaled volatile organic compounds (VOC) might be useful in the assessment of asthma. The oxidative stress results in reactive oxygen species that degrade lipids and create these compounds [46]. Two different techniques can measure exhaled VOC, including gas chromatography and the “eNose” technique (46). A recent meta-analysis suggests that evaluation of exhaled VOC could be helpfully in the diagnosis of asthma with a AUC value at 0.94 [99]. Ibrahim showed that detection of characteristic breath VOC profiles could differentiate clinically relevant disease phenotypes based on sputum inflammatory profile and asthma control [100]. In another small study in adult patients, the eNose technique could identify patients with asthma, predict which patients would lose asthma control upon withdrawal of steroid therapy, and predict which patients would respond to oral corticosteroid treatment [101]. The measurement of VOC by gas chromatography coupled with mass spectrometry could predict the risk for exacerbation in asthmatic children [102]. These promising methods need to be standardised before a plus large implementation.

### Urine metabolites

*Bromotyrosine* is formed from post-translational modification of tyrosine protein residues by hypobromous acid produced by activated eosinophils during the process of a respiratory burst [4]. It has many advantages as a potential biomarker given its stability and noninvasive detection in the urine [4]. Previous data has suggested that bromotyrosine concentrations are higher in patients with allergic asthma [103] and elevated levels of bromotyrosine are associated with airflow limitation, inadequately controlled asthma, and could predict future exacerbations [104, 105].Urinary bromotyrosine concentrations are predictive of a greater response to corticosteroids [75]. However, concordance among sputum eosinophils count, FeNO level, and urinary bromotyrosine concentration is not very high [75], so the utility of bromotyrosine in the clinical setting would probably be best when assessed as a part of a larger panel of inflammatory biomarkers [4].

*Leukotriene E*_*4*_ is a stable and product of cysteinyl leukotriene metabolism possible to measure noninvasively in urine samples [4]. Several studies have suggested that urinary leukotriene *E*_*4*_ (uLTE4) concentrations are increased in children with allergic asthma and adults with aspirin-exacerbated respiratory disease [4, 106–108]. A recent meta-analysis [109] showed that uLTE4 is a high predictive biomarker for the aspirin exacerbated respiratory disease and could potentially be used as a clinical test to identify the risk of aspirin intolerance in subjects with asthma.]Urinary LTE4 levels are increased during asthma exacerbations and correlated to the degree of airflow limitation [77]. Several data suggested that uLTE4 are increased in response to environmental tabacco smoke exposure in children and high uLTE4 levels are predictive of futures exacerbations in asthmatic children exposed to second hand smoke [110, 111]. One study showed that uLTE(4)/FeNO ratio predict a better response to montelukast than fluticasone propionate therapy in children with mild-to-moderate asthma regarding the lung function and the asthma control [112]. Another study suggested that a high uLTE4 concentration is associated with a differential response favoring ICS step-up treatment with a leukotriene receptor antagonist over long-acting β-agonists [113]. These data suggest that uLTE4 might be an important biomarker in the selection of asthma therapy [4].

### Cellular bronchial samples and bronchial biopsy

The most invasive method to study airways changes in asthmatic patients is the fiberoptic bronchoscopy with endobronchial biopsy, brushing or bronchoalveolar lavage which requires specialized medical center, training, emergency protocols and equipment [4].

The study of cellular bronchial pattern is interesting in the research. Thanks to this kind of studies, we know now that bronchial neutrophilia in bronchoalveolar lavage fluid is associated with severe asthma independent of oral corticosteroid intake [114], as well the elevated CD4+ cells expressing both IL-4 and IL-17 predicted greater asthma severity [9, 115]. Several gene signatures analysed in endobronchial brushing in the UBIOPRED cohort predicted persistent airflow limitation [116].

Usually, the bronchial biopsy is indicated in severe asthma with fixed airway obstruction secondary to airway remodelling to quantify the thickness of smooth muscle and to establish the possibility to realise a thermoplasty. Airway remodeling in severe asthma with fixed airway obstruction mainly is the consequence of the smooth muscle hypertrophy and mucosal glands hypertrophy with the increased number of fibroblasts and collagen-3 deposition within bronchial wall [117]. Thermoplasty is the first treatment which specifically targets the airway remodeling and the supposed mechanisms is the reduction of airway smooth muscle thereby reducing the airway twitchiness. Thermoplasty may be proposed as a non-pharmacological treatment in asthmatics who remained uncontrolled despite ICS. The benefice of this treatment is a reduction of exacerbation and sometimes hospitalization [117]. Several studies identified few potential biomarkers in the sputum *(MMP, Fibroblast Growth Factor-2* and *Galectin-3*) able to predict the airway remodelling with a non-invasive intervention [118–120].

## Discussion

In asthma, and particularly in the severe asthma, many biomarkers have been investigated but only few of them, so far, can be easily used in clinical practice [121]. The Table 1 summarizes the advantages, the limits and the utility in the clinical setting of major biomarkers.

**Table 1:** Summary of major biomarkers’ characteristics

Biomarker	Advantages	Limits	Utility
Blood eosinophils	-Minimal invasive -Minimal patient effort -Easy to measure and collect in the clinical setting -Correlates with sputum eosinophilia	-Painful and difficult in some patients -Varying cut-offs used to determine predictive characteristics -Can be elevated due to other causes, such as parasitic infection	-Defines the inflammatory phenotype -Predicts exacerbations, poor asthma control and greater airway obstruction -Predicts therapeutic responses to corticosteroids and biotherapies
Serum IgE	-Easy to measure -Identifies patients who may be candidates for Anti-IgE therapy	-Not predictive of response to Anti-IgE -Outperformed by other markers of T2 inflammation and allergen specific IgE	-Associated with asthma severity and airway remodelling
Serum periostin	-Marker of Il-13 activity and T2 airway inflammation	-Not currently realised in the clinical setting -Can be elevated in growing children	-Predicts a greater airway obstruction and decline of lung function -Predicts therapeutic responses to biotherapies
Sputum eosinophils	-Non invasive -Reflects the upper airways	-Difficult to collect -Not all patients can provide adequate samples -Not universally available -Requires specialized training, equipment, laboratory	-Defines the inflammatory phenotype -Predicts responses to corticosteroids and biotherapies
FeNO	-Non invasive -Minimal patient effort -Easy to collect in the clinical setting	-Multiple confounders -Requires specialized equipment	-Identifies airways inflammation -Predicts exacerbations and airways hyperreactivity -Predicts responses to corticosteroids and several biotherapies

An ideal biomarker should be suitable to identify the disease as well the specific endotype/phenotype, useful in the monitoring of the disease and to determine the prognosis, easily to obtain with minimum discomfort or risk to the patient [3, 4, 121].

According to the presence of assessable biomarkers of T2 mediated airway inflammation, the cluster-analysis identified several asthma phenotypes. The T2-high phenotype includes the classical allergic one (mild blood eosinophilia, high levels of FeNO, high level of serum total IgE) and the late-onset, nonallergic but highly eosinophilic one, frequently associated to chronic rhinosinusitis with nasal polyps (high FeNO but serum total IgE normal or elevated but probably with a lower etiopathogenetical importance) [1, 121]. The eosinophilic phenotype is associated with an intense production of IL-5 and IL-13. The T2-low phenotypes are more diversified and less well defined, with predominant neutrophilic airway inflammation, higher frequency of recurrent airway infections, higher prevalence of obesity and cigarette smoking. The mechanisms implicated in these phenotypes are the TNFα and IL-17 inflammatory pathways [69].

Unfortunately, at the moment, an ideal biomarker doesn’t exist and the overlap between the biomarkers is a reality. Using panels of biomarkers could improve probably the identification of asthma endotypes in the era of the precision medicine.

Other desired characteristics of a biomarker are the easiness and non-invasiveness of assessment. The development of point-of-care testing and non-invasive devices (one validated recently for the blood eosinophil count, others in study for the assessment of serum IgE and periostin) could accelerate the path leading to a precision medicine approach and clinical management of severe asthma [121].

Biomarkers, in addition to their role in defining phenotypes and endotypes may also have a predictive value for the response to biologic treatments. Serum total IgE is used in practice to verify that a patient with severe allergic asthma could be a candidate for omalizumab therapy and blood eosinophils count (usually ≥300 cells/μL) to prescribe biological agents such as anti-IL5 antibody in the eosinophilic refractory severe asthma. If in the last 10 years, only omalizumab was available, followed by mepolizumab, we will move in the next few years to a situation in which we will have to choose one monoclonal antibody among many (benralizumab, an IL-5 receptor antagonist; dupilumab, an IL-4 receptor alpha antagonist; tezepelumab, an anti-thymic stromal lymphopoietin antibody). This implies the need of more selective biomarkers (or panels of them) in order to identify the right biologic therapy for each single patient, in a more personalized and precise medicine approach to the disease treatment [2, 121].

## Conclusions

The implementation of the precision medicine in the management of asthma in clinical practice requires the detection of valid biomarkers. A variety of biomarkers have been used clinically to predict the response to steroid therapy, and in the clinical trial setting to identify patients that will respond to biologic therapies, but currently available biomarkers are limited in number and precision. At the moment, for a patient with a severe allergic asthma (high level of serum total IgE, high FeNO, normal or mild blood eosinophilia) uncontrolled despite a Step 4 or 5 treatment of GINA guideline, omalizumab seems to be the most adapted therapeutic option. If failure, another biologic therapy such as mepolizumab or reslizumab could be prescribe if blood eosinophilia (≥ 300 cells/μL, respectively ≥400 cells/μL). In the refractory eosinophilic asthma without atopic background (high blood eosinophilia, high FeNO, normal IgE), an anti-IL5 antibody seems to be the most appropriate. Macrolides could be an interesting therapeutic option for the patients with severe uncontrolled asthma with T2-low inflammatory pattern, as well the bronchial termoplasty in patients with airways remodeling.

Further research and validation of emerging biomarkers are needed to define the molecular phenotype of asthma, particularly in the non-T2 pathways, to predict outcomes and therapeutic response to more specific targeted therapies. The use of omics data from multiple platforms (transcriptomics, proteomics, or metabolomics) appears as a promising tool to obtain endotypes. Viewing the heterogeneity of asthma, to predict therapeutic response, the development of composite biomarkers from blood, urine and exhaled breath seams to be a more appropriate solution in practice.

## Abbreviations

ACOS: Asthma-chronic obstructive pulmonary disease overlap syndrome
ADEPT: Airways Disease Endotyping for Personalized Therapeutics
AHR: Airway hyperresponsiveness
AUC: Area under the curve
CCL26: Chemokine ligand 26
COPD: Chronic obstructive pulmonary disease
CXCR2: Chemokine receptor 2
EBC: Exhaled breath condensate
ECP: Eosinophil cationic protein
FeNO: Fractional Exhaled Nitric Oxide
FEV1/FVC: Forced expiratory volume in the one second/forced vital capacity ratio
GINA: Global Initiative for Asthma
hs-CRP: Serum high sensitive C-reactive protein
ICS: Inhaled corticosteroids
IgE: Immunoglobulin E
IL-13: Interleukin-13
IL-13: Interleukine-13
IL-17: Interleukine 17
IL-4: Interleukin 4
IL-4R-alpha: Inteleukine-4 receptor alpha units
IL-5: Interleukin 5
IL-6: Interleukin-6
IL-8: Interleukin-8
ILC: Innate lymphoid cell
MMP-9: Matrix metalloproteinase-9
OR: Odds ratio
RR: Rate ratio
SARP: Severe Asthma Research Program
TWEAK: Tumor necrosis factor-like weak inducer of apoptosis
UBIOPRED: Unbiased biomarkers for the prediction of respiratory disease
uLTE4: Urinary leukotriene *E*_*4*_
VOC: Exhaled volatile organic compounds
YKL-40: Human cartilage glycoprotein 39
μL: Microliter

## References

[1] Ray A, Raundhal M, Oriss TB, Ray P, Wenzel SE (2016). Current concepts of severe asthma. J Clin Invest.

[2] Chung KF. Personalised medicine in asthma: time for action. Eur Respir Rev. 2017. 10.1183/16000617.0064-2017. 10.1183/16000617.0064-2017 PMC9488697 28954768

[3] Eguiluz-Gracia I, Tay TR, Hew M, Escribese MM, Barber D, O’Hehir RE, et al. Recent developments and highlights in biomarkers in allergic diseases and asthma. Allergy. 2018; doi: 10.1111. 10.1111/all.13628 30289997

[4] Fitzpatrick AM (2015). Biomarkers of asthma and allergic airway diseases. Ann Allergy Asthma Immunol.

[5] Kelly RS, Dahlin A, McGeachie MJ, Qiu W, Sordillo J, Wan ES (2017). Asthma metabolomics and the potential for integrative omics in research and the clinic. Chest.

[6] Villaseñor A, Rosace D, Obeso D, Pérez-Gordo M, Chivato T, Barbas C (2017). Allergic asthma: an overview of metabolomic strategies leading to the identification of biomarkers in the field. Clin Exp Allergy.

[7] Price DB, Rigazio A, Campbell JD, Bleecker ER, Corrigan CJ, Thomas M (2015). Blood eosinophil count and prospective annual asthma disease burden: a UK cohort study. Lancet Respir Med.

[8] Hancox RJ, Pavord ID, Sears MR. Associations between blood eosinophils and decline in lung function among adults with and without asthma. Eur Respir J. 2018. 10.1183/13993003.02536-2017. 10.1183/13993003.02536-2017 29563173

[9] Konradsen JR, Skantz E, Nordlund B, Lidegran M, James A, Ono J (2015). Predicting asthma morbidity in children using proposed markers of Th2-type inflammation. Pediatr Allergy Immunol.

[10] Fitzpatrick AM (2016). Severe asthma in children: lessons learned and future directions. J Allergy Clin Immunol Pract.

[11] Wark PA, McDonald VM, Gibson PG (2015). Adjusting prednisone using blood eosinophils reduces exacerbations and improves asthma control in difficult patients with asthma. Respirol..

[12] Jabbal S, Lipworth BJ (2018). Blood eosinophils: the forgotten man of inhaled steroid dose titration. Clin Exp Allergy.

[13] Ortega HG, Yancey SW, Mayer B, Gunsoy NB, Keene ON, Bleecker ER (2016). Severe eosinophilic asthma treated with mepolizumab stratified by baseline eosinophil thresholds: a secondary analysis of the DREAM and MENSA studies. Lancet Respir Med.

[14] Bleecker ER, FitzGerald JM, Chanez P, Papi A, Weinstein SF, Barker P (2016). Efficacy and safety of benralizumab for patients with severe asthma uncontrolled with high-dosage inhaled corticosteroids and long-acting β2-agonists (SIROCCO): a randomised, multicentre, placebo-controlled phase 3 trial. Lancet.

[15] Castro M, Corren J, Pavord ID, Maspero J, Wenzel S, Rabe KF (2018). Dupilumab efficacy and safety in moderate-to-severe uncontrolled asthma. N Engl J Med.

[16] Bjermer L, Lemiere C, Maspero J, Weiss S, Zangrilli J, Germinaro M (2016). Reslizumab for inadequately controlled asthma with elevated blood eosinophil levels: a randomized phase 3 study. Chest.

[17] Casale TB, Chipps BE, Rosén K, Trzaskoma B, Haselkorn T, Omachi TA (2018). Response to omalizumab using patient enrichment criteria from trials of novel biologics in asthma. Allergy.

[18] Hanania NA, Wenzel S, Rosén K, Hsieh H-J, Mosesova S, Choy DF (2013). Exploring the effects of omalizumab in allergic asthma: an analysis of biomarkers in the EXTRA study. Am J Respir Crit Care Med.

[19] Humbert M, Taillé C, Mala L, Le Gros V, Just J, Molimard M, et al. Omalizumab effectiveness in patients with severe allergic asthma according to blood eosinophil count: the STELLAIR study. Eur Respir J. 2018. 10.1183/13993003.02523-2017. 10.1183/13993003.02523-2017 PMC6383600 29545284

[20] Zhang X-Y, Simpson JL, Powell H, Yang IA, Upham JW, Reynolds PN (2014). Full blood count parameters for the detection of asthma inflammatory phenotypes. Clin Exp Allergy.

[21] Wagener AH, de Nijs SB, Lutter R, Sousa AR, Weersink EJM, Bel EH (2015). External validation of blood eosinophils, FE(NO) and serum periostin as surrogates for sputum eosinophils in asthma. Thorax.

[22] Westerhof GA, Korevaar DA, Amelink M, de Nijs SB, de Groot JC, Wang J (2015). Biomarkers to identify sputum eosinophilia in different adult asthma phenotypes. Eur Respir J.

[23] Tommola M, Ilmarinen P, Tuomisto LE, Lehtimäki L, Haanpää J, Niemelä O, et al. Differences between asthma-COPD overlap syndrome and adult-onset asthma. Eur Respir J. 2017. 10.1183/13993003.02383-2016. 10.1183/13993003.02383-2016 28461298

[24] Nadif R, Siroux V, Boudier A, le Moual N, Just J, Gormand F (2016). Blood granulocyte patterns as predictors of asthma phenotypes in adults from the EGEA study. Eur Respir J.

[25] Korevaar DA, Westerhof GA, Wang J, Cohen JF, Spijker R, Sterk PJ (2015). Diagnostic accuracy of minimally invasive markers for detection of airway eosinophilia in asthma: a systematic review and meta-analysis. Lancet Respir Med.

[26] Koh GC-H, Shek LP-C, Goh DY-T, Van Bever H, Koh DS-Q (2007). Eosinophil cationic protein: is it useful in asthma? A systematic review. Respir Med.

[27] Kato M, Yamada Y, Maruyama K, Hayashi Y (2010). Serum eosinophil cationic protein and 27 cytokines/chemokines in acute exacerbation of childhood asthma. Int Arch Allergy Immunol.

[28] Spahn JD, Covar RA, Jain N, Gleason M, Shimamoto R, Szefler SJ (2006). Effect of montelukast on peripheral airflow obstruction in children with asthma. Ann Allergy Asthma Immunol.

[29] Grayson MH, Feldman S, Prince BT, Patel PJ, Matsui EC, Apter AJ (2018). Advances in asthma in 2017: mechanisms, biologics, and genetics. J Allergy Clin Immunol.

[30] Lopez-Guisa JM, Powers C, File D, Cochrane E, Jimenez N, Debley JS (2012). Airway epithelial cells from asthmatic children differentially express proremodeling factors. J Allergy Clin Immunol.

[31] Corren J, Lemanske RF, Hanania NA, Korenblat PE, Parsey MV, Arron JR (2011). Lebrikizumab treatment in adults with asthma. N Engl J Med.

[32] Simpson JL, Yang IA, Upham JW, Reynolds PN, Hodge S, James AL (2016). Periostin levels and eosinophilic inflammation in poorly-controlled asthma. BMC Pulm Med.

[33] Anderson HM, Lemanske RF, Arron JR, Holweg CTJ, Rajamanickam V, Gangnon RE (2017). Relationships among aeroallergen sensitization, peripheral blood eosinophils, and periostin in pediatric asthma development. J Allergy Clin Immunol.

[34] Semprini R, Caswell-Smith R, Fingleton J, Holweg C, Matthews J, Weatherall M (2017). Longitudinal variation of serum periostin levels in adults with stable asthma. J Allergy Clin Immunol.

[35] Takahashi K, Meguro K, Kawashima H, Kashiwakuma D, Kagami S-I, Ohta S (2018). Serum periostin levels serve as a biomarker for both eosinophilic airway inflammation and fixed airflow limitation in well-controlled asthmatics. J Asthma.

[36] James A, Janson C, Malinovschi A, Holweg C, Alving K, Ono J (2017). Serum periostin relates to type-2 inflammation and lung function in asthma: data from the large population-based cohort Swedish GA(2)LEN. Allergy.

[37] Kanemitsu Y, Matsumoto H, Izuhara K, Tohda Y, Kita H, Horiguchi T (2013). Increased periostin associates with greater airflow limitation in patients receiving inhaled corticosteroids. J Allergy Clin Immunol.

[38] Kanemitsu Y, Ito I, Niimi A, Izuhara K, Ohta S, Ono J (2014). Osteopontin and periostin are associated with a 20-year decline of pulmonary function in patients with asthma. Am J Respir Crit Care Med.

[39] Tajiri T, Matsumoto H, Gon Y, Ito R, Hashimoto S, Izuhara K (2016). Utility of serum periostin and free IgE levels in evaluating responsiveness to omalizumab in patients with severe asthma. Allergy.

[40] Planagumà A, Kazani S, Marigowda G, Haworth O, Mariani TJ, Israel E (2008). Airway lipoxin A4 generation and lipoxin A4 receptor expression are decreased in severe asthma. Am J Respir Crit Care Med.

[41] Levy BD, Bonnans C, Silverman ES, Palmer LJ, Marigowda G, Israel E (2005). Diminished lipoxin biosynthesis in severe asthma. Am J Respir Crit Care Med.

[42] Ono E, Dutile S, Kazani S, Wechsler ME, Yang J, Hammock BD (2014). Lipoxin generation is related to soluble epoxide hydrolase activity in severe asthma. Am J Respir Crit Care Med.

[43] Barnig C, Cernadas M, Dutile S, Liu X, Perrella MA, Kazani S (2013). Lipoxin A4 regulates natural killer cell and type 2 innate lymphoid cell activation in asthma. Sci Transl Med.

[44] Bhavsar PK, Levy BD, Hew MJ, Pfeffer MA, Kazani S, Israel E (2010). Corticosteroid suppression of lipoxin A4 and leukotriene B4 from alveolar macrophages in severe asthma. Respir Res.

[45] Barnig C, Levy BD (2013). Lipoxin A4: a new direction in asthma therapy?. Expert Rev Clin Immunol.

[46] Medrek SK, Parulekar AD, Hanania NA. Predictive biomarkers for asthma therapy. Curr Allergy Asthma Rep. 2017. 10.1007/s11882-017-0739-5. 10.1007/s11882-017-0739-5 28929293

[47] Burrows B, Martinez FD, Halonen M, Barbee RA, Cline MG (1989). Association of asthma with serum IgE levels and skin-test reactivity to allergens. N Engl J Med.

[48] Fitzpatrick AM, Teague WG, Meyers DA, Peters SP, Li X, Li H (2011). Heterogeneity of severe asthma in childhood: confirmation by cluster analysis of children in the National Institutes of Health/National Heart, Lung, and Blood Institute severe asthma research program. J Allergy Clin Immunol.

[49] Sears MR, Burrows B, Flannery EM, Herbison GP, Hewitt CJ, Holdaway MD (1991). Relation between airway responsiveness and serum IgE in children with asthma and in apparently normal children. N Engl J Med.

[50] Sherrill DL, Lebowitz MD, Halonen M, Barbee RA, Burrows B (1995). Longitudinal evaluation of the association between pulmonary function and total serum IgE. Am J Respir Crit Care Med.

[51] Bousquet J, Rabe K, Humbert M, Chung KF, Berger W, Fox H (2007). Predicting and evaluating response to omalizumab in patients with severe allergic asthma. Respir Med.

[52] James AJ, Reinius LE, Verhoek M, Gomes A, Kupczyk M, Hammar U (2016). Increased YKL-40 and Chitotriosidase in asthma and chronic obstructive pulmonary disease. Am J Respir Crit Care Med.

[53] Wang J, Lv H, Luo Z, Mou S, Liu J, Liu C, et al. Plasma YKL-40 and NGAL are useful in distinguishing ACO from asthma and COPD. Respir Res. 2018. 10.1186/s12931-018-0755-6. 10.1186/s12931-018-0755-6 PMC5870925 29580282

[54] Gomez JL, Yan X, Holm CT, Grant N, Liu Q, Cohn L, et al. Characterisation of asthma subgroups associated with circulating YKL-40 levels. Eur Respir J. 2017. 10.1183/13993003.00800-2017. 10.1183/13993003.00800-2017 PMC5967238 29025889

[55] Tang H, Fang Z, Sun Y, Li B, Shi Z, Chen J (2010). YKL-40 in asthmatic patients, and its correlations with exacerbation, eosinophils and immunoglobulin E. Eur Respir J.

[56] Guerra S, Halonen M, Sherrill DL, Venker C, Spangenberg A, Carsin A (2013). The relation of circulating YKL-40 to levels and decline of lung function in adult life. Respir Med.

[57] Silkoff PE, Laviolette M, Singh D, FitzGerald JM, Kelsen S, Backer V (2017). Identification of airway mucosal type 2 inflammation by using clinical biomarkers in asthmatic patients. J Allergy Clin Immunol.

[58] Portelli MA, Moseley C, Stewart CE, Postma DS, Howarth P, Warner JA (2017). Airway and peripheral urokinase plasminogen activator receptor is elevated in asthma, and identifies a severe, nonatopic subset of patients. Allergy.

[59] Midyat L, Gulen F, Karaca E, Ozkinay F, Tanac R, Demir E (2016). MicroRNA expression profiling in children with different asthma phenotypes. Pediatr Pulmonol.

[60] Monadi M, Firouzjahi A, Hosseini A, Javadian Y, Sharbatdaran M, Heidari B (2016). Serum C-reactive protein in asthma and its ability in predicting asthma control, a case-control study. Casp J Intern Med.

[61] Shimoda T, Obase Y, Kishikawa R, Iwanaga T (2015). Serum high-sensitivity C-reactive protein can be an airway inflammation predictor in bronchial asthma. Allergy Asthma Proc.

[62] Naik SP, A M P, S J B, Madhunapantula SV, Jahromi SR, Yadav MK (2017). Evaluation of inflammatory markers interleukin-6 (IL-6) and matrix metalloproteinase-9 (MMP-9) in asthma. J Asthma..

[63] Liu H-C, Lu M-C, Lin Y-C, Wu T-C, Hsu J-Y, Jan M-S (2014). Differences in IL-8 in serum and exhaled breath condensate from patients with exacerbated COPD or asthma attacks. J Formos.

[64] Moore WC, Hastie AT, Li X, Li H, Busse WW, Jarjour NN (2014). Sputum neutrophil counts are associated with more severe asthma phenotypes using cluster analysis. J Allergy Clin Immunol.

[65] Loza MJ, Djukanovic R, Chung KF, Horowitz D, Ma K, Branigan P (2016). Validated and longitudinally stable asthma phenotypes based on cluster analysis of the ADEPT study. Respir Res.

[66] Kuo C-HS, Pavlidis S, Loza M, Baribaud F, Rowe A, Pandis I, et al. T-helper cell type 2 (Th2) and non-Th2 molecular phenotypes of asthma using sputum transcriptomics in U-BIOPRED. Eur Respir J. 2017. 10.1183/13993003.02135-2016. 10.1183/13993003.02135-2016 28179442

[67] Baines KJ, Simpson JL, Wood LG, Scott RJ, Fibbens NL, Powell H (2014). Sputum gene expression signature of 6 biomarkers discriminates asthma inflammatory phenotypes. J Allergy Clin Immunol.

[68] Simpson JL, Scott R, Boyle MJ, Gibson PG (2006). Inflammatory subtypes in asthma: assessment and identification using induced sputum. Respirol.

[69] Ray A, Kolls JK (2017). Neutrophilic inflammation in asthma and association with disease severity. Trends Immunol.

[70] Simpson JL, Powell H, Boyle MJ, Scott RJ, Gibson PG (2008). Clarithromycin targets neutrophilic airway inflammation in refractory asthma. Am J Respir Crit Care Med.

[71] Gibson PG, Yang IA, Upham JW, Reynolds PN, Hodge S, James AL (2017). Effect of azithromycin on asthma exacerbations and quality of life in adults with persistent uncontrolled asthma (AMAZES): a randomised, double-blind, placebo-controlled trial. Lancet.

[72] Nair P, Ochkur SI, Protheroe C, Radford K, Efthimiadis A, Lee NA (2013). Eosinophil peroxidase in sputum represents a unique biomarker of airway eosinophilia. Allergy.

[73] Demarche SF, Schleich FN, Paulus VA, Henket MA, Van Hees TJ, Louis RE (2017). Asthma control and sputum eosinophils: a longitudinal study in daily practice. J Allergy Clin Immunol Pract.

[74] Smith SG, Chen R, Kjarsgaard M, Huang C, Oliveria J-P, O’Byrne PM (2016). Increased numbers of activated group 2 innate lymphoid cells in the airways of patients with severe asthma and persistent airway eosinophilia. J Allergy Clin Immunol.

[75] Cowan DC, Taylor DR, Peterson LE, Cowan JO, Palmay R, Williamson A (2015). Biomarker-based asthma phenotypes of corticosteroid response. J Allergy Clin Immunol.

[76] Petsky HL, Cates CJ, Lasserson TJ, Li AM, Turner C, Kynaston JA (2012). A systematic review and meta-analysis: tailoring asthma treatment on eosinophilic markers (exhaled nitric oxide or sputum eosinophils). Thorax.

[77] Green RH, Brightling CE, McKenna S, Hargadon B, Parker D, Bradding P (2002). Asthma exacerbations and sputum eosinophil counts: a randomised controlled trial. Lancet.

[78] Chung KF, Wenzel SE, Brozek JL, Bush A, Castro M, Sterk PJ (2014). International ERS/ATS guidelines on definition, evaluation and treatment of severe asthma. Eur Respir J.

[79] Haldar P, Brightling CE, Hargadon B, Gupta S, Monteiro W, Sousa A (2009). Mepolizumab and exacerbations of refractory eosinophilic asthma. N Engl J Med.

[80] Castro M, Mathur S, Hargreave F, Boulet L-P, Xie F, Young J (2011). Reslizumab for poorly controlled, eosinophilic asthma: a randomized, placebo-controlled study. Am J Respir Crit Care Med.

[81] Wenzel S, Ford L, Pearlman D, Spector S, Sher L, Skobieranda F (2013). Dupilumab in persistent asthma with elevated eosinophil levels. N Engl J Med.

[82] Gonem S, Berair R, Singapuri A, Hartley R, Laurencin MFM, Bacher G (2016). Fevipiprant, a prostaglandin D2 receptor 2 antagonist, in patients with persistent eosinophilic asthma: a single-centre, randomised, double-blind, parallel-group, placebo-controlled trial. Lancet Respir Med.

[83] Maes T, Cobos FA, Schleich F, Sorbello V, Henket M, De Preter K (2016). Asthma inflammatory phenotypes show differential microRNA expression in sputum. J Allergy Clin Immunol.

[84] Iwamoto H, Gao J, Koskela J, Kinnula V, Kobayashi H, Laitinen T (2014). Differences in plasma and sputum biomarkers between COPD and COPD-asthma overlap. Eur Respir J.

[85] Kim SY, Kim JD, Sol IS, Kim MJ, Kim MN, Hong JY (2018). Sputum TWEAK expression correlates with severity and degree of control in non-eosinophilic childhood asthma. Pediatr Allergy Immunol.

[86] Horváth I, Hunt J, Barnes PJ, Alving K, Antczak A, Baraldi E (2005). Exhaled breath condensate: methodological recommendations and unresolved questions. Eur Respir J.

[87] Aldakheel FM, Thomas PS, Bourke JE, Matheson MC, Dharmage SC, Lowe AJ (2016). Relationships between adult asthma and oxidative stress markers and pH in exhaled breath condensate: a systematic review. Allergy.

[88] Antczak A, Kurmanowska Z, Kasielski M, Nowak D (2000). Inhaled glucocorticosteroids decrease hydrogen peroxide level in expired air condensate in asthmatic patients. Respir Med.

[89] Dweik RA, Boggs PB, Erzurum SC, Irvin CG, Leigh MW, Lundberg JO (2011). An official ATS clinical practice guideline: interpretation of exhaled nitric oxide levels (FENO) for clinical applications. Am J Respir Crit Care Med.

[90] Kortekaas Krohn I, Shikhagaie MM, Golebski K, Bernink JH, Breynaert C, Creyns B (2018). Emerging roles of innate lymphoid cells in inflammatory diseases: clinical implications. Allergy.

[91] Dupont LJ, Rochette F, Demedts MG, Verleden GM (1998). Exhaled nitric oxide correlates with airway hyperresponsiveness in steroid-naive patients with mild asthma. Am J Respir Crit Care Med.

[92] Malinovschi A, Fonseca JA, Jacinto T, Alving K, Janson C (2013). Exhaled nitric oxide levels and blood eosinophil counts independently associate with wheeze and asthma events in National Health and nutrition examination survey subjects. J Allergy Clin Immunol.

[93] Price DB, Buhl R, Chan A, Freeman D, Gardener E, Godley C (2018). Fractional exhaled nitric oxide as a predictor of response to inhaled corticosteroids in patients with non-specific respiratory symptoms and insignificant bronchodilator reversibility: a randomised controlled trial. Lancet Respir Med.

[94] Essat M, Harnan S, Gomersall T, Tappenden P, Wong R, Pavord I (2016). Fractional exhaled nitric oxide for the management of asthma in adults: a systematic review. Eur Respir J.

[95] Calhoun WJ, Ameredes BT, King TS, Icitovic N, Bleecker ER, Castro M (2012). Comparison of physician-, biomarker-, and symptom-based strategies for adjustment of inhaled corticosteroid therapy in adults with asthma: the BASALT randomized controlled trial. JAMA.

[96] McNicholl DM, Stevenson M, McGarvey LP, Heaney LG (2012). The utility of fractional exhaled nitric oxide suppression in the identification of nonadherence in difficult asthma. Am J Respir Crit Care Med.

[97] Pavord ID, Korn S, Howarth P, Bleecker ER, Buhl R, Keene ON (2012). Mepolizumab for severe eosinophilic asthma (DREAM): a multicentre, double-blind, placebo-controlled trial. Lancet Lond Engl.

[98] Castro M, Wenzel SE, Bleecker ER, Pizzichini E, Kuna P, Busse WW (2014). Benralizumab, an anti-interleukin 5 receptor α monoclonal antibody, versus placebo for uncontrolled eosinophilic asthma: a phase 2b randomised dose-ranging study. Lancet Respir Med.

[99] Rufo JC, Madureira J, Fernandes EO, Moreira A (2016). Volatile organic compounds in asthma diagnosis: a systematic review and meta-analysis. Allergy.

[100] Ibrahim B, Basanta M, Cadden P, Singh D, Douce D, Woodcock A (2011). Non-invasive phenotyping using exhaled volatile organic compounds in asthma. Thorax.

[101] van der Schee MP, Palmay R, Cowan JO, Taylor DR (2013). Predicting steroid responsiveness in patients with asthma using exhaled breath profiling. Clin Exp Allergy J Br Soc Allergy Clin Immunol.

[102] Robroeks CM, van Berkel JJ, Jöbsis Q, van Schooten F-J, Dallinga JW, Wouters EF (2013). Exhaled volatile organic compounds predict exacerbations of childhood asthma in a 1-year prospective study. Eur Respir J.

[103] Wedes SH, Khatri SB, Zhang R, Wu W, Comhair SAA, Wenzel S (2009). Noninvasive markers of airway inflammation in asthma. Clin Transl Sci.

[104] Comhair SAA, Ricci KS, Arroliga M, Lara AR, Dweik RA, Song W (2005). Correlation of systemic superoxide dismutase deficiency to airflow obstruction in asthma. Am J Respir Crit Care Med.

[105] Wedes SH, Wu W, Comhair SAA, McDowell KM, DiDonato JA, Erzurum SC (2011). Urinary bromotyrosine measures asthma control and predicts asthma exacerbations in children. J Pediatr.

[106] Bochenek G, Kuschill-Dziurda J, Szafraniec K, Plutecka H, Szczeklik A, Nizankowska-Mogilnicka E (2014). Certain subphenotypes of aspirin-exacerbated respiratory disease distinguished by latent class analysis. J Allergy Clin Immunol.

[107] Bochenek G, Stachura T, Szafraniec K, Plutecka H, Sanak M, Nizankowska-Mogilnicka E (2018). Diagnostic accuracy of urinary LTE4 measurement to predict aspirin-exacerbated respiratory disease in patients with asthma. J Allergy Clin Immunol Pract.

[108] Rabinovitch N, Zhang L, Gelfand EW (2006). Urine leukotriene E4 levels are associated with decreased pulmonary function in children with persistent airway obstruction. J Allergy Clin Immunol.

[109] Hagan JB, Laidlaw TM, Divekar R, O’Brien EK, Kita H, Volcheck GW (2017). Urinary leukotriene E4 to determine aspirin intolerance in asthma: a systematic review and meta-analysis. J Allergy Clin Immunol Pract.

[110] Lang JE, Dozor AJ, Holbrook JT, Mougey E, Krishnan S, Sweeten S (2013). Biologic mechanisms of environmental tobacco smoke in children with poorly controlled asthma: results from a multicenter clinical trial. J Allergy Clin Immunol Pract.

[111] Rabinovitch N, Reisdorph N, Silveira L, Gelfand EW (2011). Urinary leukotriene E_4_ levels identify children with tobacco smoke exposure at risk for asthma exacerbation. J Allergy Clin Immunol.

[112] Rabinovitch N, Graber NJ, Chinchilli VM, Sorkness CA, Zeiger RS, Strunk RC (2010). Urinary leukotriene E4/exhaled nitric oxide ratio and montelukast response in childhood asthma. J Allergy Clin Immunol.

[113] Rabinovitch N, Mauger DT, Reisdorph N, Covar R, Malka J, Lemanske RF (2014). Predictors of asthma control and lung function responsiveness to step 3 therapy in children with uncontrolled asthma. J Allergy Clin Immunol.

[114] Alam R, Good J, Rollins D, Verma M, Chu H, Pham T-H (2017). Airway and serum biochemical correlates of refractory neutrophilic asthma. J Allergy Clin Immunol.

[115] Irvin C, Zafar I, Good J, Rollins D, Christianson C, Gorska MM (2014). Increased frequency of dual-positive TH2/TH17 cells in bronchoalveolar lavage fluid characterizes a population of patients with severe asthma. J Allergy Clin Immunol.

[116] Hekking P-P, Loza MJ, Pavlidis S, De Meulder B, Lefaudeux D, Baribaud F, et al. Transcriptomic gene signatures associated with persistent airflow limitation in patients with severe asthma. Eur Respir J. 2017. 10.1183/13993003.02298-2016. 10.1183/13993003.02298-2016 28954779

[117] Schleich F, Demarche S, Louis R (2016). Biomarkers in the Management of Difficult Asthma. Curr Top Med Chem.

[118] Vignola AM, Chanez P, Siena L, Chiappara G, Bonsignore G, Bousquet J (1998). Airways remodelling in asthma. Pulm Pharmacol Ther.

[119] Bissonnette ÉY, Madore A-M, Chakir J, Laviolette M, Boulet L-P, Hamid Q (2014). Fibroblast growth factor-2 is a sputum remodeling biomarker of severe asthma. J Asthma Off J Assoc Care Asthma.

[120] Mauri P, Riccio AM, Rossi R, Di Silvestre D, Benazzi L, De Ferrari L (2014). Proteomics of bronchial biopsies: galectin-3 as a predictive biomarker of airway remodelling modulation in omalizumab-treated severe asthma patients. Immunol Lett.

[121] Canonica GW, Ferrando M, Baiardini I, Puggioni F, Racca F, Passalacqua G (2018). Asthma: personalized and precision medicine. Curr Opin Allergy Clin Immunol.

